# Development and evaluating multimarker models for guiding treatment decisions

**DOI:** 10.1186/s12911-018-0619-5

**Published:** 2018-06-28

**Authors:** Parvin Tajik, Mohammad Hadi Zafarmand, Aeilko H. Zwinderman, Ben W. Mol, Patrick M. Bossuyt

**Affiliations:** 1 Department of Pathology, Department of Clinical Epidemiology, Biostatistics & Bioinformatics, Department of Obstetrics & Gynaecology, Academic Medical Centre - University of Amsterdam, Room J1b-210, PO Box 22700, 1100 DE Amsterdam, the Netherlands; 2 Department of Clinical Epidemiology, Biostatistics & Bioinformatics, Department of Obstetrics & Gynaecology, Academic Medical Centre, Amsterdam, the Netherlands; 3 Department of Clinical Epidemiology, Biostatistics & Bioinformatics, Academic Medical Centre, Amsterdam, the Netherlands; 4 Department of Obstetrics and Gynaecology, Monash University, Clayton, VIC Australia

**Keywords:** Treatment selection, Biomarker, Randomised controlled trials, Individualised medicine, Stratified medicine, Subgroup analysis, Prediction models, Prognostic, Model development, Validation

## Abstract

**Background:**

Despite the growing interest in developing markers for predicting treatment response and optimizing treatment decisions, an appropriate methodology to identify, combine and evaluate such markers has been slow to develop. We propose a step-by-step strategy for analysing data from existing randomised trials with the aim of identifying a multi-marker model for guiding decisions about treatment.

**Methods:**

We start with formulating the treatment selection problem, continue with defining the treatment threshold, prepare a list of candidate markers, develop the model, apply the model to estimate individual treatment effects, and evaluate model performance in the study group of patients who meet the trial eligibility criteria. In this process, we rely on some well-known techniques for multivariable prediction modelling, but focus on predicting benefit from treatment, rather than outcome itself. We present our approach using data from a randomised trial in which 808 women with multiple pregnancy were assigned to cervical pessary or control, to prevent adverse perinatal outcomes. Overall, cervical pessary did not reduce the risk of adverse perinatal outcomes.

**Results:**

The treatment threshold was zero. We had a preselected list of 5 potential markers and developed a logistic model including the markers, treatment and all marker-by-treatment interaction terms. The model was well calibrated and identified 35% (95% confidence interval (CI) 32 to 39%) of the trial participants as benefitting from pessary insertion. We estimated that the risk of adverse outcome could be reduced from 13.5 to 8.1% (5.4% risk reduction; 95% CI 2.1 to 8.6%) through model-based selective pessary insertion. The next step is external validation upon existence of independent trial data.

**Conclusions:**

We suggest revisiting existing trials data to explore whether differences in treatment benefit can be explained by differences in baseline characteristics of patients. This could lead to treatment selection tools which, after validation in comparable existing trials, can be introduced into clinical practice for guiding treatment decisions in future patients.

## Background

The main goal of most randomised clinical trials is inference about the effects of treatment, typically in terms of effectiveness, efficacy, or toxicity. If a statistically significant treatment effect is found, this does not imply that all eligible patients benefit from the treatment under investigation. Similarly, a failure to find such an effect does not necessarily mean that no patients would have a better outcome if treated.

Spear and colleagues [1] have analysed the efficacy of major drugs for a number of therapeutic areas, based on published data. They found that the percentage of responding patients is between 80% for Cox-2 inhibitors and 25% for cancer chemotherapy, with many of the drugs falling within the 50 to 75% response range. The safety of treatment options also varies between drugs and diseases. Exploring this heterogeneity could result in a better understanding of the underlying factors.

Most clinical trials assemble an extensive collection of baseline information on the study participants. This includes all kinds of patient characteristics, clinical findings, laboratory test and imaging results. We believe that these features, markers and test results can be used to explore the variability in the magnitude and direction of treatment benefit. Consequently, an algorithm could be developed for predicting which of the investigated treatment options is better given a specific profile. If validated, such algorithms would be able to guide treatment decisions for individual patients.

Subgroup analyses are a common strategy for investigating the heterogeneity of treatment effect, and 40 to 65% of randomised clinical trials report such analyses [2]. In conventional subgroup analysis patients are assigned to categories based on potentially influential characteristics [3]. This classification is typically performed for each characteristic separately, one at a time, and associations between the marker and the effect of treatment are evaluated by testing for marker-treatment interactions. As such, typical subgroup analyses do not account for the fact that patients have multiple characteristics, each potentially affecting the expected magnitude of a treatment benefit.

Below we propose a systematic approach to combine marker information to form combinations of markers for predicting the benefit for treatment and guiding decisions. We illustrate our approach with a model for predicting the benefits from pessary insertion in women with multiple gestation. Our framework applies techniques from multivariable prediction modelling, but we not focus on predicting outcome, but on the benefit from treatment, defined as the predicted difference in outcomes, between the two forms of treatment.

## Empirical example data

We will introduce our framework with an example. The most serious risk of multiple pregnancy (twin or triplet) is spontaneous preterm delivery, which is associated with increased perinatal mortality and short-term and long-term morbidity [4, 5]. Several measures have been considered and evaluated for minimizing this risk, including the prophylactic insertion of a cervical pessary. In the ProTwin trial, 813 consenting women with multiple gestations were randomly allocated to either pessary insertion in the first trimester or not using a pessary [6, 7]. The primary outcome measure in the trial was the occurrence of one or more adverse perinatal outcomes. Our rational to choose ProTwin trial was it’s simplicity of concept and that it was rich in terms of marker information.

## Methods

In our description, we focus on a binary primary outcome measure to simplify the exposition, but the method can also be extended to accommodate other types of outcomes.

### Step 1: Defining the treatment decision

The initial step is to carefully consider the treatment selection problem: the treatment, the comparator, the eligible patients for whom the treatment should be chosen, and the relevant outcomes. Typically, one would only consider treatment if there is a benefit (a difference in outcome after treatment) and, in most cases, the benefit must be large enough to outweigh the harms, burden or costs of treatment.

In some trials, the primary outcome measure captures all of the main consequences of treatment decision, both positive and negative, and a difference in the primary outcome measure between the trial arms would indicate a benefit from treatment. An example are trials with mortality as the primary outcome measure, where mortality could be reduced by effective treatment but also increased because of the morbidity from that very same treatment.

If the primary outcome measure does not capture the full range of benefits and harms, but only a benefit, a treatment threshold can be defined. This treatment threshold is the extent of treatment benefit at which treatment would outweigh the negative consequences that are not captured in the primary outcome measure, such as harms and treatment burden. In this context, initiation of treatment is only justified when the treatment effect, as expressed in the difference in the primary outcome, is larger than the treatment threshold.

In scenarios where the treatment under investigation has adverse effects, one would set a treatment threshold above zero: the treatment effect should be large enough to justify the negative consequences. A zero treatment threshold would imply that any benefit from treatment justifies administration.

### Step 2: Preparing the list of candidate markers

The next step is developing a list of markers that are believed to have potential for predicting the benefit for patients from treatment. The benefit of treatment is defined as a difference in outcome, comparing treatment versus no treatment, this comes down to a prediction of (differential) treatment outcomes.

Knowledge of the treatment’s underlying mechanism of action can help in defining relevant markers. Another source for identifying markers is reviewing literature which have reported on markers as risk factors of the outcome, as factors prognostic of response to treatment, as factors for subgroup analysis, or as factors that interact with treatment. Such markers have potential to be useful for predicting the benefit from treatment.

### Step 3: Data sources

The data would ideally come from a randomised clinical trial. In randomised trials associations between marker with treatment outcome and treatment benefit can be studied without bias, since randomisation in sufficiently large trials guarantees exchangeability. The quality of data collection in randomised trials is usually good as well, even though data collection is not typically done for studying associations with treatment benefit. In a pragmatic trial, the trial study group is typically representative of the population who could qualify for treatment, with minimal exclusion criteria.

The methods described in this study may also be generalised to data from observational studies, however, treatment allocation in observational studies is not independent of the baseline characteristics. In this setting the investigator would be well-advised to stratify on variables that are potentially associated with treatment provision and outcome [8].

### Step 4: Developing a prediction model including marker-by-treatment interaction terms

A reasonably sized list of markers should be selected for consideration. The number of markers that can be reliably investigated in a dataset depends on the size of the dataset, and whether the dataset includes enough number of outcomes. This needs a cautious selection of markers for modelling treatment benefit, where a common rule of thumb is to require at least 5 to 10 events per predictor under investigation [9, 10].

The next step is selecting a modelling type. Several statistical methods are proposed for combining markers for treatment selection including using generalised linear regression modelling [11–20], classification trees [14, 21] or directly maximizing the mean outcome under marker-based treatment [12]. For a binary outcome we propose logistic regression to model the outcome of treatment as a function of the specified markers and treatment, including an interaction between each marker and treatment. We base this recommendation on simulation studies done by Kang and colleagues [14]. They showed that other, more sophisticated methods did not result in a marker combination that performed better than the selection identified with logistic regression. Pepe and colleagues [22] also found logistic regression to be remarkably robust in the classification context.

The investigator may choose to include all the pre-specified markers in the model. In other cases, a variable selection procedure can be considered to reduce the number of markers in the model. Several techniques are available for variable selection and model building in usual prediction modelling setting [9, 10, 23–28]. Some of these have been specifically designed for the context of treatment selection [13, 15, 16]. An exhaustive discussion of the pros and cons is beyond the scope of this paper.

There is extensive literature showing that predictions from multivariable models can be improved for future subjects if such predictions are shrunk towards the average using a shrinkage factor. Several techniques are available for obtaining a shrinkage factor, including split-sample, cross-validation or bootstrapping [9, 10].

Bootstrapping is the preferred method, certainly when the development sample is relatively small and /or a high number of candidate predictors is studied [26]. Some penalised regression methods such as the least absolute shrinkage and selection operator (LASSO) might also be attractive, since they perform both variable selection and shrinkage [29]. One can use penalty terms for main effects and interactions or set LASSO on interaction terms [30].

### Step 5: Evaluating benefit

Next, we want to evaluate how well the model performs in improving treatment selection. The starting point for this process is an estimation of the treatment benefit for each participant in the trial, which is the difference between the counterfactual outcomes with and without treatment. As is well known, we can never observe a treatment benefit in individual patients, but under the usual assumptions, such as exchangeability, we can evaluate the average treatment benefit in identifiable subgroups, based on the identified markers. In this context, the treatment benefit for an individual patient can be calculated as the difference between the estimated risk of an adverse outcome without treatment and the estimated risk with treatment. If the treatment threshold, defined in step one, has a value above zero, one should subtract the treatment threshold from the calculated difference in probabilities to estimate the treatment benefit. We then can visualize the estimated treatment benefits by two approaches. One is depicting the distribution of estimated treatment benefit in a histogram. This plot visualizes the range and variability of the estimated benefit [8, 31].

#### Calibration assessment

Calibration refers to the agreement between observed outcomes and predictions. Calibration of a treatment selection model can be specifically investigated by plotting the average observed treatment benefit against the average expected treatment benefit in evenly sized groups defined by ranges of predicted treatment benefits, based on deciles, for example. Ideally, if the observed effects and predicted effects agree over the whole range of probability differences, the plot will show a diagonal line. Calibration around the decision point of zero is particularly important because miscalibration at this point could result in change of treatment decision. Since the calibration of the model for treatment benefit is related to the calibration of the logistic regression model itself, one can also plot calibration plots for the calculated risk of outcome itself, with and without treatment.

### Step 6: Model performance and population impact

The next step is measuring to what extent the developed model can affect the choice of treatment, and how many adverse events could be prevented if the model was used for treatment selection. The following summary measures are proposed for this purpose.

#### Multimarker positivity rate

The first key summary measure is the proportion of patients identified by the model as benefiting from treatment (treatment benefit above treatment threshold). We call this the multimarker positivity rate, to make it comparable with single marker investigation literature [8, 32]. This identifies the proportion of the patients in whom the treatment would be recommended. In contexts where the standard strategy is treating everyone and model is developed to identify those who do not benefit (enough) from the treatment, the marker negativity rate identifies the proportion of patients for whom the treatment recommendation would change.

#### Average benefit of treatment in multimarker positives

This measure evaluates the treatment effect in the subgroup of patients who are multimarker positive. It is the difference in adverse event rate in the multimarker positive patients who were not treated versus multimarker positives actually treated in the trial.

#### Average benefit of no treatment in multimarker negatives

In the same way, we can calculate the effect of avoiding treatment in the subgroup of patients who are multimarker negative. This measure is the difference in adverse event rate in the multimarker negatives who were treated versus negatives who were not treated.

#### Change in outcome with a model-based strategy

Based on the previous measures, we can calculate the estimated change in the outcome in the target population, if treatment decisions are guided by the multimarker model. It is an estimate of the population impact of using the model to select treatment or the reduction in the risk of outcome in population by application of the model-based strategy. This measure, or a variation, has been advocated by many as the global measure of marker performance [8, 12, 15, 16, 20, 32–34]. We assume that under a model-based strategy, all multimarker positives are treated and all multimarker negatives avoid treatment. In scenarios where the default strategy is treating everybody, this measure of population impact is calculated by multiplying the multimarker negativity rate with the average benefit of no treatment in multimarker negatives [8].

### Step 7: External validation

It is not enough to demonstrate a reasonable or good performance of a model on the development sample only. In those circumstances most models show optimistic results, even after optimism corrections [9, 23, 24, 27]. It is essential to confirm that a developed model predicts well in similar but different individuals, outside the development sample.

One option is using datasets of other existing trials, wherein patients sampled from the target population have been randomly allocated to treatment strategies comparable to those in the development trial. Validation is only possible if the validation dataset includes data on model markers and on the outcome of interest. One can use the markers and assigned weights (regression coefficients) of the original model, predict the treatment benefit for each patient included in the validation trial, and study the distribution of benefit, calibration, and estimate the population impact of using the treatment selection model.

Validation of the performance of the model can also be done in a new trial, wherein patients are randomly assigned to a control group, which undergoes the default treatment strategy, and an intervention group, where predictions are made available to individuals and/or healthcare professionals to guide decision-making. This would result in a real-world estimate of the population impact of the model.

## Results

Using the ProTwin trial data, we illustrate here the proposed step-by-step strategy for analysing data from an existing randomised trial with the aim of identifying a multi-marker model for guiding decisions about treatment.

### Step 1: Defining the treatment decision

In our example, there is no intervention that can prevent preterm delivery and its consequences in women with multiple gestation, so the comparator is no treatment [35–39]. The side effects associated with pessary are not serious and include vaginal discharge and pain. The device itself is not expensive (€38 per pessary) and insertion can be done in an outpatient clinic by a gynaecologist. So, the associated harms and costs are rather low in comparison with the adverse outcomes it might be able to reduce. This allows us to define a treatment threshold of zero, which means that one would opt for pessary insertion with any chance of benefit. The ProTwin trial showed that cervical pessary insertion could not significantly reduce the risk in the study group [6]. The question then is whether there is an identifiable subgroup of women who would benefit from pessary insertion.

### Step 2: Preparing the list of candidate markers

The exact mechanism of action of cervical pessary insertion is unknown. Pessaries surround the cervix and therefore might prevent preterm birth through mechanically supporting the cervix. Based on this hypothesis, cervical length could be a relevant marker, since it can measure the extent of cervical strength and, as such, the need for external support with a pessary. Other possible markers are those previously reported as risk factors of preterm birth in multiple gestation: obstetric history [40], previous history of preterm delivery [41], whether the twins share the placenta [42], and number of foetuses (twins versus triplet) [43].

### Step 3: Data sources

The ProTwin trial was a population based study with a relatively broad set of inclusion criteria, which allowed us to study all relevant marker treatment associations; only women whose fetus(es) had major fetal abnormalities or placenta previa were excluded.

### Step 4: Developing a prediction model including marker-by-treatment interaction terms

We used logistic regression with the full model approach, and included all a priori selected candidate markers in the multivariable analyses, without any further predictor selection. This avoids the so-called predictor selection bias and overfitting [26]. We chose to make a full model because we had a rather small number of markers to investigate, and all of these markers were relatively easy to obtain, without further costs. The shrinkage factor we calculated for our model by randomly drawing 200 bootstrap samples of the ProTwin data was 0.76. Table 1 presents the estimated regression coefficients of the model, after multiplication by the shrinkage factor. Among the specified markers monochorionicity (interaction odds ratio; OR_int_ = 0.30) and short cervix (OR_int_ = 0.36) were associated with a differential benefit from pessary insertion, while history of previous preterm birth (OR_int_ = 14.01) and triplet pregnancy (OR_int_ = 3.67) were associated with an increased risk of adverse outcomes after pessary insertion.

**Table 1:** Estimated regression coefficients and corresponding odds ratios (95% confidence interval) of the treatment selection model

Predictor	Shrunken Beta^a^	OR (95% CI)
Intercept	−2.21	
Main terms		
Pessary	0.22	1.25 (0.62–2.52)
Short cervix	1.07	2.92 (1.36–6.26)
Monochorionic	1.21	3.35 (1.79–6.28)
Parous with no previous preterm birth	−0.83	0.44 (0.22–0.87)
Parous with at least one previous preterm birth	−1.46	0.23 (0.03–1.82)
Triplet	0.57	1.77 (0.33–9.36)
Interaction terms		
Pessary × Short cervix	−1.01	0.36 (0.13–1.03)
Pessary × Monochorionic	−1.22	0.30 (0.12–0.76)
Pessary × Parous with no previous preterm birth	0.54	1.72 (0.66–4.48)
Pessary × Parous with at least one previous preterm birth	2.64	14.01 (1.50–130.9)
Pessary × Triplet	1.30	3.67 (0.42–32.32)

^a^Shrunken with an average shrinkage factor of 0.76

### Step 5: Evaluating benefit

For each of the ProTwin trial participants we used the final model in Table 1 two times. First, we calculated the probability of adverse outcome with pessary and again estimated the probability of adverse outcome without pessary. We then subtracted the probability with pessary from the probability without pessary. In our example, the treatment threshold was set at zero, therefore there was no need to modify the estimated treatment benefit.

**Fig. 1:**
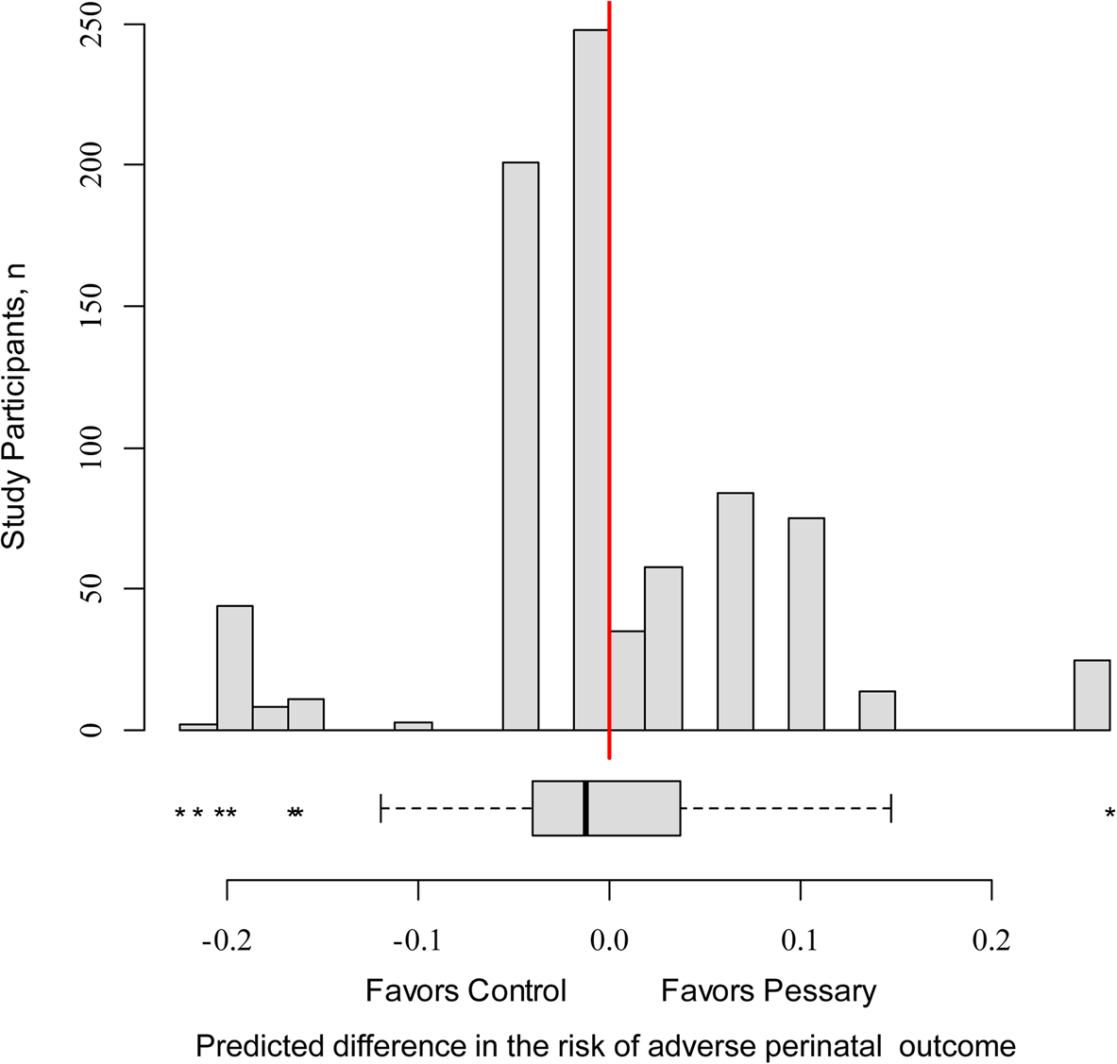
Distribution of the estimated treatment benefit in ProTwin trial participants

Figure 1 shows the distribution of the estimated benefit from pessary in participants of ProTwin trial. The wide range of estimated treatment benefit in the study participants shows the extent of heterogeneity in treatment effect, predicted by the model. It also illustrates how large the subpopulations are that would benefit from pessary, or be harmed by it.

#### Calibration assessment

**Fig. 2:**
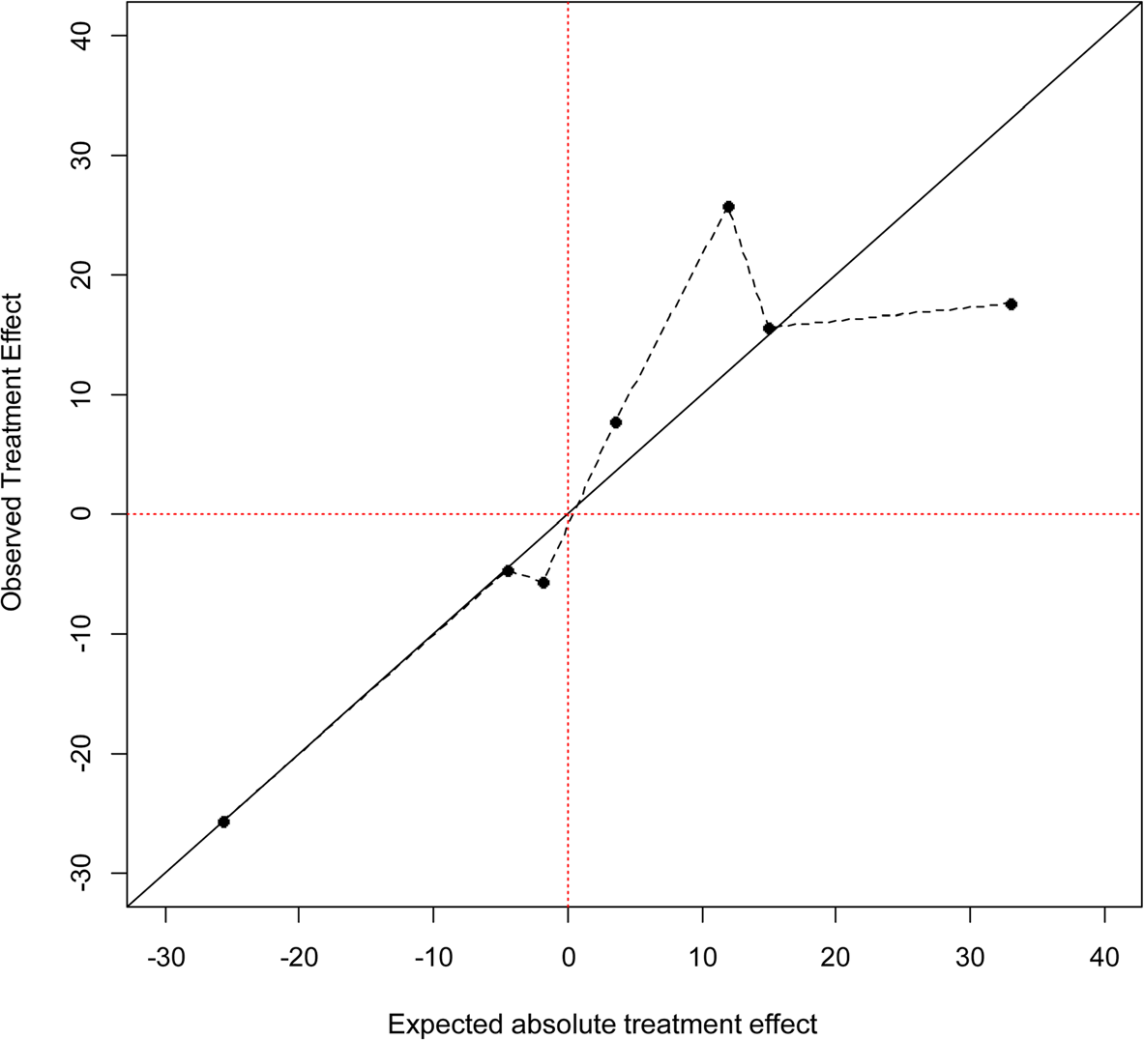
Plot assessing calibration of estimated treatment benefits from the model

Figure 2 displays the calibration of the model we developed in ProTwin participants. Predictions are on the x-axis and observations are on the y-axis. In each group, predictions are simply the average of estimated treatment benefits of the individuals in that group; observations are the result of subtracting the proportion of patients allocated to control with an adverse outcome in that group from the comparable proportion from those allocated to pessary insertion in the same group.

### Step 6: Model performance and population impact

We used the following summary measures to show to what extent the developed model can affect the choice of treatment, and how many adverse events could be prevented if the model was used for treatment selection.

#### Multimarker positivity rate

Our example model identified 35% of women in the ProTwin trial as benefitting from pessary insertion. Considering that the default management in women with multiple gestation is no treatment, this measure also indicates that for 35% of the patient population the recommended treatment would change, if guided by the model.

#### Average benefit of treatment in multimarker positives

In the subgroup of women identified by our model as those benefiting from pessary, the average benefit was a 15% reduction in the risk of adverse outcome (95% CI: 6 to 24%).

#### Average benefit of no treatment in multimarker negatives

In ProTwin trial among the subgroup of women identified by our model as those not benefitting from pessary, the average reduction in the risk of adverse outcome was 8.2% (95% CI: 2 to 12%). This means that by avoiding unnecessary pessary insertion, we could reduce the risk of adverse events in the subgroup of multimarker negatives with 8.2%.

#### Change in outcome with a model-based strategy

In our example the standard strategy is no intervention in all women, thus the improvement from implementing the model-based strategy equals the risk reduction in multimarker positives who would receive treatment. This is estimated by multiplying the multimarker positivity rate with the average benefit of treatment in multimarker positives (35% × 15.4% = 5.4%; 95% CI 2.1 to 8.6%). As the risk of adverse outcome with the default strategy is 13.5% (obtained from the control arm of the ProTwin trial) then the risk can be reduced to 8.1% (13,5% minus 5.4%) through selective pessary insertion.

### Step 7: External validation

ProTwin trial was the first trial evaluating the use of a pessary in women with multiple gestations. There does not yet exist another trial dataset allowing validation. Some recently initiated trials could offer suitable validation options for our model.

## Discussion

We have presented here a framework for developing and evaluating multimarker models for guiding treatment decisions. The methods combine classical techniques for multivariable modelling with a decision-making perspective, focusing on benefit, the difference in counterfactual outcomes, related to a choice between interventions, not just on the outcome after treatment. Although several elements of the approach are well studied, there are still areas in need of further development.

The literature on statistical methods for combining markers is quite extensive [9, 10, 22–24, 26, 27], but the vast majority of articles have focused on combining markers for predicting outcome without treatment, or under a single treatment [14]. Yet the risk of an outcome does not translate immediately into a benefit; benefit is defined as the difference in outcome. Patients at high risk of an adverse outcome are not necessarily able to reduce that risk by undergoing treatment, while those at low risk may still benefit, further reducing their risk.

Others have also argued that modelling benefit, rather than outcome, can be a superior approach for identifying variables that can guide treatment decisions. Claggett and colleagues [44] have shown that the best performing risk models, based on associations between markers and outcome in each treatment group, do not necessarily produce the best performing model of a treatment effect. Such a strategy may miss markers that are strongly associated with the treatment effect but have modest main effects, and risks including markers that have strong main effects but modest interactions with treatment. In the study by Kang and colleagues [14, 45], fitting a risk model to each treatment group separately tended to produce marker combinations with inferior performance, compared to those that simultaneously considered both treatment groups. This study showed that applying logistic regression with including marker treatment interactions tend to produce marker combinations with a superior performance compared to a few other techniques.

Other approaches for guiding treatment decisions based on multivariable modelling have also been proposed. Dorresteijn and colleagues predicted the effect of rosuvastatin on cardiovascular events for individual patients using data from a randomised trial [46]. They developed a multivariable model including covariate-treatment interactions. Using the resulting model, they predicted each patient’s cardiovascular outcome, with and without rosuvastatin. The estimated absolute risk reduction achieved by rosuvastatin treatment was then calculated as the difference between the two risk predictions, aggregated over all trial participants. For evaluation of the performance of the model they calculated the net benefit described by Vickers and colleagues [47]. They also proposed plotting the net benefit for various strategies at different treatment thresholds in a decision curve [47–49].

Other strategies for evaluating differences in treatment effect have been proposed. Cai and colleagues [19], for example, have considered a systematic two-stage estimation procedure for individual-level treatment differences for treatment selection in future patients. Their method can be applied to randomised trials for comparing two treatments. In the first stage, parametric or semiparametric models are fitted 3separately to each treatment group to estimate individual-level differences. An index score system is then calculated from these differences for grouping individuals. The average treatment difference is consistently estimated within each stratum of the index using a nonparametric function estimation method. There is an optimal treatment for individuals belonging to strata in which the average treatment difference is significantly different from zero.

Foster and colleagues [17] proposed a two-stage method as well, called Virtual Twins. In the first stage, regression forest techniques are used to estimate the patient-specific event probabilities with and without treatment (twins). In the second stage, the subpopulation of patients who experienced enhanced treatment benefit are defined by regression trees. The authors quantify the enhanced treatment effect in the subgroup as the difference between the absolute risk reduction by treatment in the subgroup and the average treatment effect in the overall population.

Kovalchik and colleagues [50] proposed a framework based on a proportional interactions model, where all treatment-marker interaction terms were assumed to be proportional to the main effects. Dusseldorp and Van Mechelen [21] derived partitioning algorithms, called qualitative interaction trees, resulting in a binary tree to identify qualitative treatment-by-marker interactions. At each partitioning step, the treatment effect difference between subgroups, as well as the subgroup sizes, were considered to refine the subpopulations.

Overall, a broad range of methods have now been investigated for combining the marker information to identify subset of patients who would benefit from treatments. It is quite challenging to compare the methods, since the aims of the procedures vary from one class of methods to the other and there is still no consensus on the measure of evaluation of performance.

As a final step, treatment selection models can be transformed into instruments for clinical use, wherein model-based predictions are presented as a web-based app, or applications for smart phones and tablets. They could also be embedded in electronic patient records as decision aid tools.

## Conclusion

We believe that the framework, as presented here, is a simple roadmap, which could offer valuable perspectives for developing individualised decision support tools, and for generating new hypotheses. As such, it fits nicely in the well-received suggestions for developing and strengthening stratified and personalised medicine. The benefits of a more refined approach for making treatment recommendations will not only materialize from novel predictive markers and companion diagnostics, but also from the careful combination of existing markers and patient characteristics, as advocated here.

### Note

All summary measures presented in this section and their corresponding confidence intervals can be estimated using the R package TreatmentSelection [8], which is publicly available (https://cran.r-project.org/web/packages/TreatmentSelection/index.html).

## Abbreviations

CI: Confidence interval
LASSO: Least absolute shrinkage and selection operator
OR: Odds ratio
OR_int_: Odds ratio for interaction
